# Crystal structure of methyl 6-meth­oxy-11-(4-meth­oxy­phen­yl)-16-methyl-14-phenyl-8,12-dioxa-14,15-di­aza­tetra­cyclo­[8.7.0.0^2,7^.0^13,17^]hepta­deca-2(7),3,5,13(17),15-penta­ene-10-carboxyl­ate

**DOI:** 10.1107/S1600536814017929

**Published:** 2014-08-09

**Authors:** V. Vinayagam, M. Bakthadoss, S. Murugavel, N. Manikandan

**Affiliations:** a Department of Organic Chemistry, University of Madras, Maraimalai Campus, Chennai 600 025, India; b Department of Chemistry, Pondicherry University, Puducherry 605 014, India; c Department of Physics, Thanthai Periyar Government Institute of Technology, Vellore 632 002, India; d Department of Physics, Bharathidasan Engineering College, Nattrampalli, Vellore 635 854, India

**Keywords:** crystal structure, conformation, crystal packing, chromene

## Abstract

In the title compound, C_30_H_28_N_2_O_6_, the pyran ring adopts a slightly distorted half-chair conformation and the pyrone ring adopts an envelope conformation, with the C atom bearing the carboxyl­ate group as the flap. The pyrazole ring [maximum deviation = 0.002 (2) Å] forms a dihedral angle of 13.2 (1)° with the attached benzene ring. The near-planar atoms of the pyran ring and the pyrazole ring are close to coplanar, the dihedral angles between their mean planes being 6.4 (1)°. The dihedral angle between the pyrone ring and the benzene ring of the chromene unit is 10.7 (1)°. The mol­ecular conformation is stabilized by an intra­molecular C—H⋯O hydrogen bond, which generates an *S*(6) ring motif. In the crystal, C—H⋯O inter­actions generate supra­molecular chains propagating in [100] and these are connected into double layers that stack along the *c*-axis direction by weak π–π inter­actions between pyrazole rings [centroid–centroid distance = 3.801 (1) Å].

## Abstract 2

In the title compound, the pyran and pyrone rings adopt slightly distorted half-chair and envelope conformations, respectively. In the crystal, C—H⋯O and π–π inter­actions connect the mol­ecules, forming double layers that stack along the *c*-axis direction.

## Chemical context   

Chromenes are components of many natural products (Ellis & Lockhart, 2007 ▶) and incorporated in numerous medicinal drugs as significant chromophores. They have shown to display anti­viral, anti­tumoral, anti-anaphylactic, spasmolytic, diuretic and clotting activity (Horton *et al.*, 2003 ▶). Furthermore, they can be used as photo-active materials, biodegradable agrochemicals and pigments. As part of our studies in this area, the crystal structure of the title compound has been determined and the results are presented here.
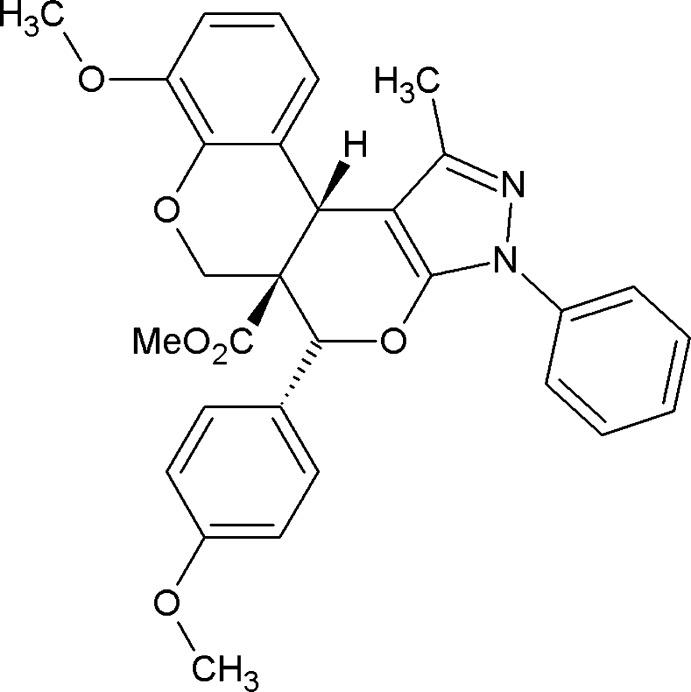


## Structural commentary   

**Figure 1:**
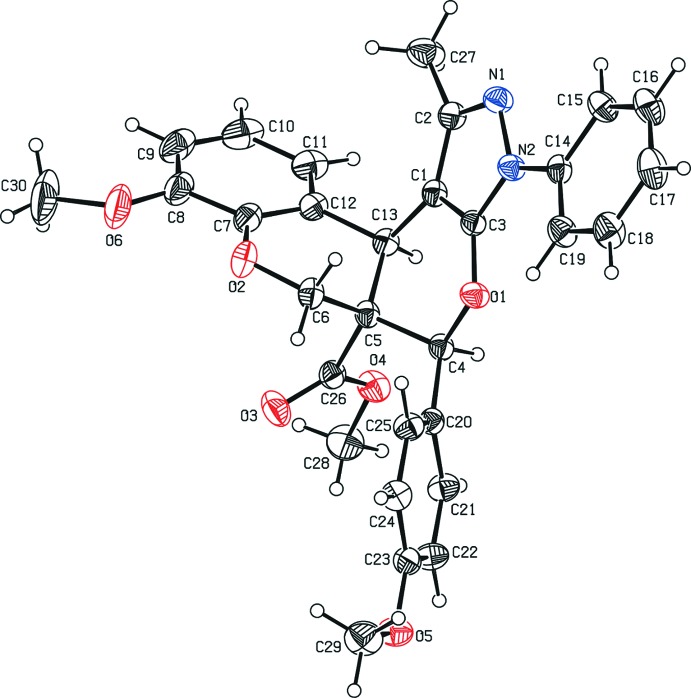
The mol­ecular structure of the title compound, showing displacement ellipsoids at the 30% probability level.

Fig. 1 ▶ shows a displacement ellipsoid plot of the title compound, with the atom-numbering scheme. The pyran ring (O1/C1/C3/C4/C5/C13) adopts a slightly distorted half-chair conformation, with the local twofold rotation axis running through the mid-points of bonds C3—C1 and C5—C4 [asymmetry parameter (Duax *et al.*, 1976 ▶) Δ*C*_2_[C3–C1] = 7.5 (2)°] The pyrone ring (O2/C5/C6/C7/C12/C13) adopts an envelope conformation, with the C5 [displacement = 0.347 (1) Å] atom as the flap and with puckering parameters *q*_2_ = 0.3973 (2) Å and ϕ_2_ = 119.7 (2)°. The pyrazole ring is approximately planar, with a maximum deviation of 0.002 (2) Å for atom C2, and forms a dihedral angle of 13.2 (1)° with the attached benzene ring. The planar atoms of the pyran ring and the pyrazole ring are close to coplanar, the dihedral angles between their mean planes being 6.4 (1)°. Moreover, the planar atoms of the pyrone ring and the benzene ring of the chromene unit are also almost coplanar, the dihedral angle between their mean planes being 10.7 (1)°. The geometric parameters of the title mol­ecule agree well with those reported for similar structures (Kanchanadevi *et al.*, 2013*a* ▶,*b* ▶).

## Supra­molecular features   

**Figure 2:**
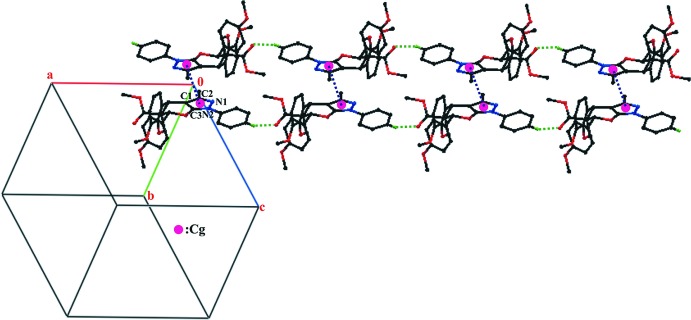
A view of stacking of supra­molecular double layer along the *c* axis. The C—H⋯O and π—π inter­actions are shown as green and blue dotted lines, respectively.

The mol­ecular conformation is stabilized by an intra­molecular C19—H19⋯O1 hydrogen bond, which generates an *S*(6) ring motif. The crystal packing features C17—H17⋯O3 hydrogen bonds, which form a supra­molecular chain along the *a* axis. This chain is connected into double layer that stacks along the *c* axis (Table 1 ▶ and Fig. 2 ▶; *Cg* is the centroid of the pyrazole N1/N2/C3/C1/C2 ring) by π–π inter­actions, with *Cg*⋯*Cg* ^ii^ = 3.801 (1) Å [symmetry code: (ii) −*x*, −*y*, −*z*].

## Database survey   

The title compound, (I), is closely related to 16-methyl-11-(2-methyl­phen­yl)-14-phenyl-8,12-dioxa-14,15-di­aza­tetra­cyclo[8.7.0.02,7.013,17]hepta­deca-2(7),3,5,13 (17),15-penta­ene-10-carbo­nitrile, (II) (Kanchanadevi *et al.*, 2013*a* ▶), and methyl 11,14,16-triphenyl-8,12-dioxa-14,15-di­aza­tetra­cyclo[8.7.0.0^2,7^.0^13,17^]hepta­deca-2(7),3,5,13 (17),15-penta­ene-10-carboxyl­ate, (III) (Kanchanadevi *et al.*, 2013*b* ▶). The pyran and pyrone rings of (II) and (III) adopt half-chair conformations, while the pyran and pyrone rings of (I) adopt half-chair and envelope conformations, respectively. The pyrazole ring forms dihedral angles of 13.2 (1), 16.9 (1) and 15.1 (1)°, respectively, for (I), (II) and (III) with the attached benzene ring.

## Synthesis and crystallization   

A mixture of (*E*)-methyl 2-[(2-formyl-6-meth­oxy­phen­oxy)meth­yl]-3-(4-meth­oxy­phen­yl)acrylate (0.356g, 1mmol) and 3-methyl-1-phenyl-1*H*-pyrazol-5-one (0.174 g, 1 mmol) was placed in a round-bottomed flask and melted at 453 K for 1 h. After completion of the reaction as indicated by thin-layer chromatography, the crude product was washed with 5 ml of an ethyl acetate and hexane mixture (1:49 ratio), which successfully provided the title compound as a colourless solid in 93% yield. Colourless blocks were obtained by slow evaporation of an ethyl acetate solution at room temperature.

## Refinement   

Crystal data, data collection and structure refinement details are summarized in Table 2 ▶. All the H atoms were positioned geometrically, with C—H = 0.93–0.98 Å, and constrained to ride on their parent atom, with *U*_iso_(H) = 1.5*U*_eq_(C) for methyl H atoms and 1.2*U*_eq_(C) for other H atoms. Owing to poor agreement, the reflections 100, 011 and 100 were omitted from the final cycles of refinement.

## Supplementary Material

Crystal structure: contains datablock(s) global, I. DOI: 10.1107/S1600536814017929/hb7257sup1.cif

Structure factors: contains datablock(s) I. DOI: 10.1107/S1600536814017929/hb7257Isup2.hkl

Supporting information file. DOI: 10.1107/S1600536814017929/hb7257Isup3.cml

CCDC reference: 962784

Additional supporting information:  crystallographic information; 3D view; checkCIF report

## Figures and Tables

**Table 1:** Hydrogen-bond geometry (Å, °) *Cg* is the centroid of the N1/N2/C3/C1/C2 ring pyrazole.

*D*—H⋯*A*	*D*—H	H⋯*A*	*D*⋯*A*	*D*—H⋯*A*
C19—H19⋯O1	0.93	2.28	2.907 (2)	124
C17—H17⋯O3^i^	0.93	2.54	3.433 (2)	161

Symmetry code: (i) 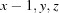.

**Table 2:** Experimental details

Crystal data	
Chemical formula	C_30_H_28_N_2_O_6_
*M*_r_	512.54
Crystal system, space group	Monoclinic, *P*2_1_/*c*
Temperature (K)	293
*a*, *b*, *c* (Å)	12.9549 (5), 14.5280 (5), 13.8522 (4)
β (°)	100.433 (2)
*V* (Å^3^)	2564.00 (15)
*Z*	4
Radiation type	Mo *K*α
μ (mm^−1^)	0.09
Crystal size (mm)	0.23 × 0.21 × 0.15

Data collection	
Diffractometer	Bruker APEXII CCD
Absorption correction	Multi-scan (*SADABS*; Bruker, 2004 ▶)
*T*_min_, *T*_max_	0.979, 0.986
No. of measured, independent and observed [*I* > 2σ(*I*)] reflections	23615, 4509, 3508
*R*_int_	0.032
(sin θ/λ)_max_ (Å^−1^)	0.594

Refinement	
*R*[*F* ^2^ > 2σ(*F* ^2^)], *wR*(*F* ^2^), *S*	0.037, 0.098, 1.02
No. of reflections	4509
No. of parameters	348
H-atom treatment	H-atom parameters constrained
Δρ_max_, Δρ_min_ (e Å^−3^)	0.19, −0.14

Computer programs: *APEX2* and *SAINT* (Bruker, 2004 ▶), *SHELXS97* and *SHELXL97* (Sheldrick, 2008 ▶), *ORTEP-3 for Windows* (Farrugia, 2012 ▶) and *PLATON* (Spek, 2009 ▶).

## supplementary crystallographic information

### Crystal data

**Table:**

C_30_H_28_N_2_O_6_	*Z* = 4
*M**_r_* = 512.54	*F*(000) = 1080
Monoclinic, *P*2_1_/*c*	*D*_x_ = 1.328 Mg m^−^^3^
Hall symbol: -P 2ybc	Mo *K*α radiation, λ = 0.71073 Å
*a* = 12.9549 (5) Å	θ = 2.0–25.0°
*b* = 14.5280 (5) Å	µ = 0.09 mm^−^^1^
*c* = 13.8522 (4) Å	*T* = 293 K
β = 100.433 (2)°	Block, colourless
*V* = 2564.00 (15) Å^3^	0.23 × 0.21 × 0.15 mm

### Data collection

**Table:**

Bruker APEXII CCD diffractometer	4509 independent reflections
Radiation source: fine-focus sealed tube	3508 reflections with *I* > 2σ(*I*)
Graphite monochromator	*R*_int_ = 0.032
Detector resolution: 10.0 pixels mm^-1^	θ_max_ = 25.0°, θ_min_ = 2.4°
ω scans	*h* = −15→15
Absorption correction: multi-scan (*SADABS*; Bruker, 2004)	*k* = −17→17
*T*_min_ = 0.979, *T*_max_ = 0.986	*l* = −16→16
23615 measured reflections	

### Refinement

**Table:**

Refinement on *F*^2^	Secondary atom site location: difference Fourier map
Least-squares matrix: full	Hydrogen site location: inferred from neighbouring sites
*R*[*F*^2^ > 2σ(*F*^2^)] = 0.037	H-atom parameters constrained
*wR*(*F*^2^) = 0.098	*w* = 1/[σ^2^(*F*_o_^2^) + (0.046*P*)^2^ + 0.5491*P*] where *P* = (*F*_o_^2^ + 2*F*_c_^2^)/3
*S* = 1.02	(Δ/σ)_max_ < 0.001
4509 reflections	Δρ_max_ = 0.19 e Å^−^^3^
348 parameters	Δρ_min_ = −0.14 e Å^−^^3^
0 restraints	Extinction correction: *SHELXL97* (Sheldrick, 2008), Fc^*^=kFc[1+0.001xFc^2^λ^3^/sin(2θ)]^-1/4^
Primary atom site location: structure-invariant direct methods	Extinction coefficient: 0.0075 (7)

### Special details

**Table:**

Geometry. All esds (except the esd in the dihedral angle between two l.s. planes) are estimated using the full covariance matrix. The cell esds are taken into account individually in the estimation of esds in distances, angles and torsion angles; correlations between esds in cell parameters are only used when they are defined by crystal symmetry. An approximate (isotropic) treatment of cell esds is used for estimating esds involving l.s. planes.
Refinement. Refinement of F^2^ against ALL reflections. The weighted R-factor wR and goodness of fit S are based on F^2^, conventional R-factors R are based on F, with F set to zero for negative F^2^. The threshold expression of F^2^ > 2sigma(F^2^) is used only for calculating R-factors(gt) etc. and is not relevant to the choice of reflections for refinement. R-factors based on F^2^ are statistically about twice as large as those based on F, and R- factors based on ALL data will be even larger.

### Fractional atomic coordinates and isotropic or equivalent isotropic displacement parameters (Å^2^)

**Table:**

	*x*	*y*	*z*	*U*_iso_*/*U*_eq_	
C1	0.09417 (11)	0.03532 (10)	0.11857 (10)	0.0359 (3)	
C2	0.04588 (12)	−0.04204 (10)	0.15296 (11)	0.0409 (4)	
C3	0.01793 (11)	0.10082 (10)	0.10840 (10)	0.0352 (3)	
C4	0.11683 (11)	0.20846 (10)	0.03943 (10)	0.0359 (3)	
H4	0.1064	0.1824	−0.0269	0.043*	
C5	0.21498 (11)	0.16198 (10)	0.10138 (10)	0.0355 (3)	
C6	0.23039 (12)	0.19093 (12)	0.20849 (10)	0.0444 (4)	
H6A	0.2417	0.2569	0.2126	0.053*	
H6B	0.1667	0.1777	0.2334	0.053*	
C7	0.34317 (12)	0.05975 (13)	0.24446 (12)	0.0482 (4)	
C8	0.43098 (14)	0.02194 (16)	0.30561 (14)	0.0637 (5)	
C9	0.46352 (15)	−0.06504 (17)	0.28691 (17)	0.0747 (7)	
H9	0.5201	−0.0915	0.3286	0.090*	
C10	0.41272 (16)	−0.11336 (15)	0.20656 (18)	0.0703 (6)	
H10	0.4351	−0.1724	0.1945	0.084*	
C11	0.32897 (14)	−0.07494 (13)	0.14395 (14)	0.0564 (5)	
H11	0.2971	−0.1070	0.0884	0.068*	
C12	0.29210 (12)	0.01174 (11)	0.16377 (11)	0.0429 (4)	
C13	0.20139 (11)	0.05619 (10)	0.09589 (10)	0.0367 (3)	
H13	0.2030	0.0363	0.0286	0.044*	
C14	−0.16950 (11)	0.10535 (11)	0.13707 (10)	0.0388 (4)	
C15	−0.25161 (13)	0.04791 (12)	0.14825 (12)	0.0492 (4)	
H15	−0.2415	−0.0154	0.1532	0.059*	
C16	−0.34859 (14)	0.08532 (15)	0.15202 (14)	0.0613 (5)	
H16	−0.4039	0.0468	0.1596	0.074*	
C17	−0.36458 (14)	0.17851 (15)	0.14481 (14)	0.0636 (5)	
H17	−0.4302	0.2032	0.1475	0.076*	
C18	−0.28280 (14)	0.23487 (14)	0.13359 (14)	0.0603 (5)	
H18	−0.2934	0.2981	0.1284	0.072*	
C19	−0.18506 (13)	0.19915 (12)	0.12997 (12)	0.0489 (4)	
H19	−0.1299	0.2381	0.1228	0.059*	
C20	0.12462 (11)	0.31102 (10)	0.03090 (10)	0.0363 (3)	
C21	0.16048 (13)	0.34863 (11)	−0.04866 (11)	0.0454 (4)	
H21	0.1753	0.3100	−0.0979	0.055*	
C22	0.17462 (14)	0.44161 (12)	−0.05640 (12)	0.0505 (4)	
H22	0.1992	0.4654	−0.1103	0.061*	
C23	0.15246 (12)	0.50008 (11)	0.01571 (11)	0.0436 (4)	
C24	0.11484 (13)	0.46416 (11)	0.09468 (12)	0.0458 (4)	
H24	0.0986	0.5030	0.1431	0.055*	
C25	0.10136 (12)	0.37067 (11)	0.10157 (11)	0.0435 (4)	
H25	0.0760	0.3470	0.1551	0.052*	
C26	0.31011 (12)	0.19241 (11)	0.05938 (11)	0.0419 (4)	
C28	0.39686 (15)	0.18027 (16)	−0.07564 (14)	0.0697 (6)	
H28A	0.4041	0.2460	−0.0770	0.104*	
H28B	0.3848	0.1569	−0.1415	0.104*	
H28C	0.4600	0.1537	−0.0393	0.104*	
C27	0.08955 (15)	−0.13469 (12)	0.17983 (16)	0.0657 (5)	
H27A	0.0355	−0.1735	0.1967	0.099*	
H27B	0.1460	−0.1298	0.2350	0.099*	
H27C	0.1154	−0.1608	0.1252	0.099*	
C29	0.15834 (18)	0.65225 (13)	0.07959 (15)	0.0687 (6)	
H29A	0.0862	0.6527	0.0872	0.103*	
H29B	0.1789	0.7132	0.0640	0.103*	
H29C	0.2015	0.6321	0.1396	0.103*	
C30	0.58200 (18)	0.0550 (3)	0.42712 (19)	0.1291 (13)	
H30A	0.5819	−0.0029	0.4605	0.194*	
H30B	0.6081	0.1022	0.4737	0.194*	
H30C	0.6262	0.0508	0.3787	0.194*	
N2	−0.06991 (9)	0.06546 (9)	0.13442 (9)	0.0388 (3)	
N1	−0.05199 (10)	−0.02486 (9)	0.16222 (10)	0.0442 (3)	
O1	0.02331 (8)	0.18946 (7)	0.08051 (8)	0.0419 (3)	
O2	0.31621 (9)	0.14614 (9)	0.26917 (8)	0.0568 (3)	
O3	0.37641 (9)	0.24377 (9)	0.09954 (9)	0.0623 (4)	
O4	0.30904 (9)	0.15651 (8)	−0.02902 (8)	0.0519 (3)	
O5	0.17081 (10)	0.59132 (8)	0.00264 (9)	0.0579 (3)	
O6	0.47757 (11)	0.07731 (12)	0.38029 (11)	0.0908 (5)	

### Atomic displacement parameters (Å^2^)

**Table:**

	*U*^11^	*U*^22^	*U*^33^	*U*^12^	*U*^13^	*U*^23^
C1	0.0329 (8)	0.0412 (9)	0.0338 (7)	0.0008 (7)	0.0065 (6)	−0.0011 (6)
C2	0.0384 (9)	0.0407 (9)	0.0441 (8)	0.0019 (7)	0.0090 (7)	0.0006 (7)
C3	0.0326 (8)	0.0390 (9)	0.0345 (7)	−0.0017 (7)	0.0072 (6)	0.0009 (6)
C4	0.0298 (8)	0.0440 (9)	0.0349 (7)	−0.0010 (6)	0.0087 (6)	0.0002 (6)
C5	0.0298 (8)	0.0435 (9)	0.0339 (7)	−0.0001 (6)	0.0074 (6)	−0.0016 (6)
C6	0.0386 (9)	0.0556 (10)	0.0378 (8)	0.0009 (8)	0.0040 (7)	−0.0033 (7)
C7	0.0355 (9)	0.0652 (12)	0.0450 (9)	0.0047 (8)	0.0103 (7)	0.0132 (8)
C8	0.0382 (10)	0.0949 (16)	0.0569 (11)	0.0044 (10)	0.0059 (8)	0.0275 (11)
C9	0.0403 (11)	0.1043 (18)	0.0820 (15)	0.0206 (12)	0.0174 (10)	0.0461 (14)
C10	0.0525 (12)	0.0711 (14)	0.0967 (16)	0.0269 (11)	0.0385 (12)	0.0353 (12)
C11	0.0465 (10)	0.0587 (11)	0.0705 (12)	0.0118 (9)	0.0279 (9)	0.0124 (9)
C12	0.0323 (8)	0.0523 (10)	0.0468 (9)	0.0063 (7)	0.0145 (7)	0.0092 (7)
C13	0.0329 (8)	0.0442 (9)	0.0345 (7)	0.0032 (7)	0.0102 (6)	−0.0006 (6)
C14	0.0319 (8)	0.0507 (10)	0.0342 (7)	−0.0010 (7)	0.0075 (6)	−0.0035 (7)
C15	0.0392 (9)	0.0542 (11)	0.0570 (10)	−0.0073 (8)	0.0163 (8)	−0.0061 (8)
C16	0.0361 (10)	0.0796 (15)	0.0716 (12)	−0.0112 (10)	0.0185 (9)	−0.0101 (10)
C17	0.0320 (10)	0.0828 (15)	0.0763 (13)	0.0066 (10)	0.0108 (9)	−0.0153 (11)
C18	0.0422 (10)	0.0596 (12)	0.0786 (13)	0.0098 (9)	0.0101 (9)	−0.0066 (10)
C19	0.0355 (9)	0.0514 (11)	0.0607 (10)	−0.0003 (8)	0.0115 (8)	−0.0033 (8)
C20	0.0307 (8)	0.0423 (9)	0.0359 (7)	−0.0002 (6)	0.0058 (6)	0.0013 (6)
C21	0.0558 (10)	0.0470 (10)	0.0364 (8)	0.0018 (8)	0.0160 (7)	−0.0006 (7)
C22	0.0605 (11)	0.0504 (11)	0.0452 (9)	0.0001 (8)	0.0217 (8)	0.0077 (8)
C23	0.0398 (9)	0.0404 (9)	0.0498 (9)	0.0000 (7)	0.0058 (7)	0.0023 (7)
C24	0.0473 (10)	0.0459 (10)	0.0460 (9)	0.0046 (8)	0.0134 (7)	−0.0042 (7)
C25	0.0441 (9)	0.0491 (10)	0.0408 (8)	0.0017 (8)	0.0170 (7)	0.0026 (7)
C26	0.0327 (8)	0.0483 (10)	0.0448 (9)	0.0024 (7)	0.0074 (7)	0.0038 (7)
C28	0.0544 (12)	0.0998 (16)	0.0629 (12)	−0.0039 (11)	0.0324 (10)	0.0098 (11)
C27	0.0535 (12)	0.0471 (11)	0.0994 (15)	0.0047 (9)	0.0215 (11)	0.0151 (10)
C29	0.0829 (15)	0.0448 (11)	0.0787 (13)	0.0057 (10)	0.0151 (11)	−0.0064 (10)
C30	0.0450 (13)	0.251 (4)	0.0817 (17)	−0.0043 (18)	−0.0152 (12)	0.036 (2)
N2	0.0327 (7)	0.0406 (7)	0.0451 (7)	−0.0006 (6)	0.0118 (5)	0.0023 (6)
N1	0.0400 (8)	0.0414 (8)	0.0528 (8)	−0.0010 (6)	0.0124 (6)	0.0047 (6)
O1	0.0306 (6)	0.0416 (6)	0.0558 (6)	0.0013 (5)	0.0141 (5)	0.0074 (5)
O2	0.0526 (7)	0.0713 (9)	0.0406 (6)	0.0068 (6)	−0.0070 (5)	−0.0016 (6)
O3	0.0405 (7)	0.0801 (9)	0.0660 (8)	−0.0186 (7)	0.0087 (6)	−0.0069 (7)
O4	0.0471 (7)	0.0659 (8)	0.0486 (6)	−0.0057 (6)	0.0246 (5)	−0.0032 (6)
O5	0.0696 (8)	0.0412 (7)	0.0641 (8)	−0.0025 (6)	0.0155 (6)	0.0025 (6)
O6	0.0571 (9)	0.1361 (14)	0.0674 (9)	0.0008 (9)	−0.0204 (7)	0.0192 (9)

### Geometric parameters (Å, º)

**Table:**

C1—C3	1.360 (2)	C16—H16	0.9300
C1—C2	1.410 (2)	C17—C18	1.370 (3)
C1—C13	1.509 (2)	C17—H17	0.9300
C2—N1	1.3211 (19)	C18—C19	1.378 (2)
C2—C27	1.481 (2)	C18—H18	0.9300
C3—O1	1.3499 (17)	C19—H19	0.9300
C3—N2	1.3555 (18)	C20—C25	1.381 (2)
C4—O1	1.4553 (16)	C20—C21	1.384 (2)
C4—C20	1.499 (2)	C21—C22	1.370 (2)
C4—C5	1.554 (2)	C21—H21	0.9300
C4—H4	0.9800	C22—C23	1.380 (2)
C5—C6	1.5202 (19)	C22—H22	0.9300
C5—C26	1.521 (2)	C23—O5	1.3646 (19)
C5—C13	1.547 (2)	C23—C24	1.378 (2)
C6—O2	1.4239 (19)	C24—C25	1.375 (2)
C6—H6A	0.9700	C24—H24	0.9300
C6—H6B	0.9700	C25—H25	0.9300
C7—O2	1.363 (2)	C26—O3	1.1962 (19)
C7—C12	1.380 (2)	C26—O4	1.3288 (19)
C7—C8	1.402 (2)	C28—O4	1.4480 (19)
C8—O6	1.362 (3)	C28—H28A	0.9600
C8—C9	1.372 (3)	C28—H28B	0.9600
C9—C10	1.378 (3)	C28—H28C	0.9600
C9—H9	0.9300	C27—H27A	0.9600
C10—C11	1.378 (3)	C27—H27B	0.9600
C10—H10	0.9300	C27—H27C	0.9600
C11—C12	1.392 (2)	C29—O5	1.417 (2)
C11—H11	0.9300	C29—H29A	0.9600
C12—C13	1.511 (2)	C29—H29B	0.9600
C13—H13	0.9800	C29—H29C	0.9600
C14—C19	1.378 (2)	C30—O6	1.428 (3)
C14—C15	1.383 (2)	C30—H30A	0.9600
C14—N2	1.4209 (19)	C30—H30B	0.9600
C15—C16	1.379 (2)	C30—H30C	0.9600
C15—H15	0.9300	N2—N1	1.3753 (17)
C16—C17	1.370 (3)		
			
C3—C1—C2	103.54 (13)	C16—C17—H17	120.4
C3—C1—C13	121.08 (13)	C17—C18—C19	120.92 (18)
C2—C1—C13	135.36 (13)	C17—C18—H18	119.5
N1—C2—C1	111.99 (13)	C19—C18—H18	119.5
N1—C2—C27	118.48 (14)	C18—C19—C14	119.55 (16)
C1—C2—C27	129.52 (15)	C18—C19—H19	120.2
O1—C3—N2	121.81 (13)	C14—C19—H19	120.2
O1—C3—C1	128.52 (13)	C25—C20—C21	117.70 (14)
N2—C3—C1	109.64 (13)	C25—C20—C4	122.77 (13)
O1—C4—C20	106.97 (11)	C21—C20—C4	119.48 (13)
O1—C4—C5	110.95 (11)	C22—C21—C20	121.37 (15)
C20—C4—C5	114.54 (12)	C22—C21—H21	119.3
O1—C4—H4	108.1	C20—C21—H21	119.3
C20—C4—H4	108.1	C21—C22—C23	120.12 (15)
C5—C4—H4	108.1	C21—C22—H22	119.9
C6—C5—C26	108.70 (12)	C23—C22—H22	119.9
C6—C5—C13	108.45 (12)	O5—C23—C24	124.63 (15)
C26—C5—C13	111.28 (12)	O5—C23—C22	115.95 (14)
C6—C5—C4	111.63 (12)	C24—C23—C22	119.42 (15)
C26—C5—C4	107.52 (11)	C25—C24—C23	119.78 (15)
C13—C5—C4	109.27 (11)	C25—C24—H24	120.1
O2—C6—C5	113.59 (13)	C23—C24—H24	120.1
O2—C6—H6A	108.8	C24—C25—C20	121.60 (14)
C5—C6—H6A	108.8	C24—C25—H25	119.2
O2—C6—H6B	108.8	C20—C25—H25	119.2
C5—C6—H6B	108.8	O3—C26—O4	124.07 (15)
H6A—C6—H6B	107.7	O3—C26—C5	124.53 (14)
O2—C7—C12	124.17 (14)	O4—C26—C5	111.38 (13)
O2—C7—C8	115.21 (17)	O4—C28—H28A	109.5
C12—C7—C8	120.59 (18)	O4—C28—H28B	109.5
O6—C8—C9	125.34 (18)	H28A—C28—H28B	109.5
O6—C8—C7	115.3 (2)	O4—C28—H28C	109.5
C9—C8—C7	119.4 (2)	H28A—C28—H28C	109.5
C8—C9—C10	120.24 (18)	H28B—C28—H28C	109.5
C8—C9—H9	119.9	C2—C27—H27A	109.5
C10—C9—H9	119.9	C2—C27—H27B	109.5
C9—C10—C11	120.5 (2)	H27A—C27—H27B	109.5
C9—C10—H10	119.7	C2—C27—H27C	109.5
C11—C10—H10	119.7	H27A—C27—H27C	109.5
C10—C11—C12	120.1 (2)	H27B—C27—H27C	109.5
C10—C11—H11	120.0	O5—C29—H29A	109.5
C12—C11—H11	120.0	O5—C29—H29B	109.5
C7—C12—C11	119.06 (15)	H29A—C29—H29B	109.5
C7—C12—C13	119.52 (15)	O5—C29—H29C	109.5
C11—C12—C13	121.34 (15)	H29A—C29—H29C	109.5
C1—C13—C12	115.22 (12)	H29B—C29—H29C	109.5
C1—C13—C5	106.87 (11)	O6—C30—H30A	109.5
C12—C13—C5	108.98 (12)	O6—C30—H30B	109.5
C1—C13—H13	108.5	H30A—C30—H30B	109.5
C12—C13—H13	108.5	O6—C30—H30C	109.5
C5—C13—H13	108.5	H30A—C30—H30C	109.5
C19—C14—C15	119.94 (15)	H30B—C30—H30C	109.5
C19—C14—N2	121.54 (14)	C3—N2—N1	109.20 (12)
C15—C14—N2	118.51 (15)	C3—N2—C14	131.47 (13)
C16—C15—C14	119.42 (17)	N1—N2—C14	119.34 (12)
C16—C15—H15	120.3	C2—N1—N2	105.62 (12)
C14—C15—H15	120.3	C3—O1—C4	112.50 (11)
C17—C16—C15	120.90 (17)	C7—O2—C6	118.73 (12)
C17—C16—H16	119.6	C26—O4—C28	116.07 (14)
C15—C16—H16	119.6	C23—O5—C29	117.42 (14)
C18—C17—C16	119.27 (17)	C8—O6—C30	117.7 (2)
C18—C17—H17	120.4		
			
C3—C1—C2—N1	0.40 (17)	C16—C17—C18—C19	−0.3 (3)
C13—C1—C2—N1	179.16 (15)	C17—C18—C19—C14	0.4 (3)
C3—C1—C2—C27	−178.69 (17)	C15—C14—C19—C18	−0.3 (2)
C13—C1—C2—C27	0.1 (3)	N2—C14—C19—C18	−179.53 (15)
C2—C1—C3—O1	177.68 (14)	O1—C4—C20—C25	39.53 (18)
C13—C1—C3—O1	−1.3 (2)	C5—C4—C20—C25	−83.85 (17)
C2—C1—C3—N2	−0.19 (16)	O1—C4—C20—C21	−143.14 (14)
C13—C1—C3—N2	−179.17 (12)	C5—C4—C20—C21	93.47 (16)
O1—C4—C5—C6	−55.68 (16)	C25—C20—C21—C22	1.3 (2)
C20—C4—C5—C6	65.55 (16)	C4—C20—C21—C22	−176.14 (15)
O1—C4—C5—C26	−174.82 (12)	C20—C21—C22—C23	−0.4 (3)
C20—C4—C5—C26	−53.59 (16)	C21—C22—C23—O5	178.81 (15)
O1—C4—C5—C13	64.28 (14)	C21—C22—C23—C24	−0.8 (3)
C20—C4—C5—C13	−174.49 (11)	O5—C23—C24—C25	−178.58 (15)
C26—C5—C6—O2	−64.78 (17)	C22—C23—C24—C25	1.0 (2)
C13—C5—C6—O2	56.34 (17)	C23—C24—C25—C20	0.0 (2)
C4—C5—C6—O2	176.78 (12)	C21—C20—C25—C24	−1.1 (2)
O2—C7—C8—O6	−0.7 (2)	C4—C20—C25—C24	176.25 (14)
C12—C7—C8—O6	177.18 (15)	C6—C5—C26—O3	−11.5 (2)
O2—C7—C8—C9	179.50 (16)	C13—C5—C26—O3	−130.89 (16)
C12—C7—C8—C9	−2.6 (3)	C4—C5—C26—O3	109.48 (17)
O6—C8—C9—C10	−177.29 (18)	C6—C5—C26—O4	170.07 (13)
C7—C8—C9—C10	2.4 (3)	C13—C5—C26—O4	50.69 (16)
C8—C9—C10—C11	0.2 (3)	C4—C5—C26—O4	−68.94 (16)
C9—C10—C11—C12	−2.7 (3)	O1—C3—N2—N1	−178.09 (12)
O2—C7—C12—C11	177.79 (14)	C1—C3—N2—N1	−0.06 (16)
C8—C7—C12—C11	0.1 (2)	O1—C3—N2—C14	1.7 (2)
O2—C7—C12—C13	1.1 (2)	C1—C3—N2—C14	179.75 (13)
C8—C7—C12—C13	−176.62 (14)	C19—C14—N2—C3	−13.5 (2)
C10—C11—C12—C7	2.6 (2)	C15—C14—N2—C3	167.34 (15)
C10—C11—C12—C13	179.21 (15)	C19—C14—N2—N1	166.34 (14)
C3—C1—C13—C12	142.17 (14)	C15—C14—N2—N1	−12.87 (19)
C2—C1—C13—C12	−36.4 (2)	C1—C2—N1—N2	−0.43 (16)
C3—C1—C13—C5	20.94 (18)	C27—C2—N1—N2	178.76 (15)
C2—C1—C13—C5	−157.65 (16)	C3—N2—N1—C2	0.30 (15)
C7—C12—C13—C1	−93.43 (17)	C14—N2—N1—C2	−179.54 (12)
C11—C12—C13—C1	89.95 (17)	N2—C3—O1—C4	−169.78 (12)
C7—C12—C13—C5	26.64 (18)	C1—C3—O1—C4	12.6 (2)
C11—C12—C13—C5	−149.98 (14)	C20—C4—O1—C3	−168.68 (11)
C6—C5—C13—C1	72.28 (14)	C5—C4—O1—C3	−43.10 (15)
C26—C5—C13—C1	−168.19 (11)	C12—C7—O2—C6	−0.3 (2)
C4—C5—C13—C1	−49.61 (14)	C8—C7—O2—C6	177.55 (14)
C6—C5—C13—C12	−52.83 (15)	C5—C6—O2—C7	−29.7 (2)
C26—C5—C13—C12	66.70 (15)	O3—C26—O4—C28	2.2 (2)
C4—C5—C13—C12	−174.72 (11)	C5—C26—O4—C28	−179.39 (14)
C19—C14—C15—C16	0.1 (2)	C24—C23—O5—C29	5.4 (2)
N2—C14—C15—C16	179.32 (14)	C22—C23—O5—C29	−174.23 (16)
C14—C15—C16—C17	0.0 (3)	C9—C8—O6—C30	17.2 (3)
C15—C16—C17—C18	0.1 (3)	C7—C8—O6—C30	−162.52 (19)

### Hydrogen-bond geometry (Å, º)

*Cg* is the centroid of the N1/N2/C3/C1/C2 pyrazole ring.

**Table:**

*D*—H···*A*	*D*—H	H···*A*	*D*···*A*	*D*—H···*A*
C19—H19···O1	0.93	2.28	2.907 (2)	124
C17—H17···O3^i^	0.93	2.54	3.433 (2)	161

Symmetry code: (i) *x*−1, *y*, *z*.
